# Identifying sex-specific sub-phenotypes of Alzheimer's disease progression using longitudinal electronic health records

**DOI:** 10.1016/j.ebiom.2026.106391

**Published:** 2026-07-16

**Authors:** Weimin Meng, Qiang Yang, Jie Xu, Yu Huang, Cankun Wang, Qianqian Song, Lixin Song, Jiang Bian, Qin Ma, Anjun Ma, Rui Yin

**Affiliations:** a Department of Health Outcomes & Biomedical Informatics, University of Florida, Gainesville, FL, 32611, USA; b School of Medicine, Indiana University, Indianapolis, IN, 46202, USA; c Department of Biomedical Informatics, Ohio State University, Columbus, OH, 43210, USA; d School of Nursing, University of Texas Health Science Center at San Antonio, San Antonio, TX, 78229, USA

**Keywords:** Alzheimer's disease, Electronic health records, Sex-stratified analysis, Disease trajectory, Machine learning

## Abstract

**Background:**

Alzheimer's Disease (AD) is a complex neurodegenerative disorder, with women comprising nearly two-thirds of individuals with AD. However, sex-specific heterogeneity in AD progression remains insufficiently understood. A data-driven approach is needed to characterise such heterogeneity from longitudinal electronic health records (EHRs).

**Methods:**

We developed a deep learning-based framework to uncover sex-specific AD sub-phenotypes using longitudinal EHRs from OneFlorida+ Clinical Research Consortium. We constructed temporal representations of these EHRs and employed an autoencoder architecture to generate latent embeddings, followed by clustering to derive sex-specific sub-phenotypes with associated progression patterns. We also performed statistical and survival analyses to unravel the characteristics of our identified sub-phenotypes.

**Findings:**

From 1665 individuals with AD (961 females, 704 males), we identified five major sex-specific sub-phenotypes of AD with distinct progression pathways and comorbidity patterns. Female-dominant sub-phenotypes presented later AD onset, longer disease duration, and enrichment of respiratory and neurological disorders. Male-dominant sub-phenotypes exhibited earlier onset, shorter duration, and higher prevalence of endocrine and metabolic conditions. Survival analysis showed significant differences in time to AD onset across sub-phenotypes.

**Interpretation:**

Our findings revealed distinct disease trajectories and comorbidity patterns between male- and female-dominant subgroups with AD. This study provides insight into sex-specific AD progression and demonstrates a data-driven framework for characterising disease heterogeneity using longitudinal EHRs.

**Funding:**

This study was supported by grants from the 10.13039/100006827Florida Department of Health, the 10.13039/100000030Centers for Disease Control and Prevention, the 10.13039/100000066National Institute of Environmental Health Sciences, and the 10.13039/100000002NIH10.13039/100006108National Center for Advancing Translational Sciences.

**Research in context**

**Evidence before this study**

Alzheimer's disease (AD) exhibits pronounced sex disparities, as women not only represent two-thirds of all individuals with AD but also show different clinical pathways and comorbidity profiles compared with men. Previous studies have identified biological and genetic sex-specific factors, such as APOE ε4-related vulnerability and hormonal modulation of tau pathology. However, most existing studies have focused on static clinical risk factors and treated sex primarily as a covariate, leaving the role of sex in shaping heterogeneous AD progression trajectories largely unexplored. Recent work using electronic health records (EHRs) has characterised AD subtypes and disease trajectories, but these approaches rarely account for sex-specific heterogeneity or the temporal interplay between comorbid conditions and AD onset.

**Added value of this study**

Using longitudinal EHRs from the OneFlorida+ Clinical Research Consortium, we applied a deep learning–based temporal autoencoder to delineate sex-specific trajectories of AD progression. We identified five distinct sub-phenotypes reflecting divergent clinical pathways and survival patterns between men and women. Female-dominant trajectories were marked by co-occurring respiratory and neurological disorders, suggesting associations between systemic inflammation and neurocognitive factors, whereas male-dominant trajectories were enriched for metabolic and endocrine abnormalities, highlighting possible vascular–metabolic associations with cognitive decline. Across all trajectories, circulatory disorders such as hypertension and genitourinary conditions were notable. By integrating temporal comorbidity dynamics with sex-stratified modelling, this study provides a data-driven framework for characterising sex-specific AD progression patterns and identifying clinically meaningful disease associations.

**Implications of all the available evidence**

Our findings highlight that sex represents an important factor associated with heterogeneity in AD progression, interacting with clinical and biological factors to shape diverse disease trajectories. Recognising sex-stratified trajectories can facilitate earlier detection of high-risk sex subgroups. More broadly, this trajectory-based, data-driven framework offers a scalable paradigm for identifying sex-specific clinical associations across complex chronic diseases.

## Introduction

Alzheimer's disease (AD), recognised as the predominant form of dementia, is a multifaceted and progressive neurodegenerative disorder that currently affects an estimated 6.9 million Americans as of 2024,^1^ with a potential rise to 13.85 million by 2060.^2^ Compelling evidence indicates that heterogeneity exists in AD progression, with patients exhibiting considerable variability^3^ in AD progression and clinical phenotypes,^4^, ^5^, ^6^, ^7^, ^8^ which presents a challenge in understanding the disease trajectory. Sub-phenotypes define clinically meaningful subgroups of patients with AD and can improve our understanding of disease progression heterogeneity while informing personalised treatment strategies.^7^^,^^9^, ^10^, ^11^, ^12^

Basic and clinical research has indicated that sex difference is one of the most important factors associated with AD progression complexity.^13^, ^14^, ^15^, ^16^ Here, sex refers to biological characteristics, categorised as male or female. Approximately two-thirds of individuals with AD are female,^1^ and recent studies suggest significant sex differences in clinical severity,^17^, ^18^, ^19^ neuropathological characteristics,^20^ and genetic factors^14^^,^^21^ in AD. It is reported that for women aged 65, the lifetime risk of developing AD is 21.2%, about twice the risk seen in men.^1^ Regarding genetic risk, accelerated cognitive decline in females has been partially attributed to the effects of the APOE ε4 allele.^22^, ^23^, ^24^ In terms of clinical phenotypes, some studies found that women are more likely to develop multiple comorbidities, which may interact and collectively increase their risk of developing AD.^25^ Despite substantial research on sex differences in AD, the heterogeneity of sex-specific progression patterns within distinct AD sub-phenotypes remains under-investigated.

The longitudinal electronic health records (EHRs)^26^^,^^27^ consist of a wide variety of critical health events of patients through routine care, including diagnoses, medications, laboratory measurements, and other relevant clinical information, which can offer long-term insights into AD development.^28^, ^29^, ^30^ For example, Lee et al.^31^ developed deep predictive clustering on temporal phenotyping to identify distinct AD subgroups with varying disease trajectories, including those with faster cognitive decline, higher comorbidity burdens and differential responses to treatment interventions. Xu et al.^29^ proposed an outcome-oriented model using Long Short-Term Memory (LSTM)^32^ to identify progression pathways for AD onset, deriving several AD progression subtypes related to clinical phenotypes such as cardiovascular diseases. In terms of sex difference study of AD, Tang et al.^33^ performed comprehensive phenotyping and network analyses, gaining insight into clinical characteristics and sex-specific clinical associations in AD. Furthermore, Tang et al.^34^ demonstrated how EHRs and knowledge networks can be leveraged for AD onset prediction from different index times and uncover sex-specific biological insights. However, existing approaches often overlook how sex influences the long-term progression trajectories throughout the course of AD.^12^

**Fig. 1:**
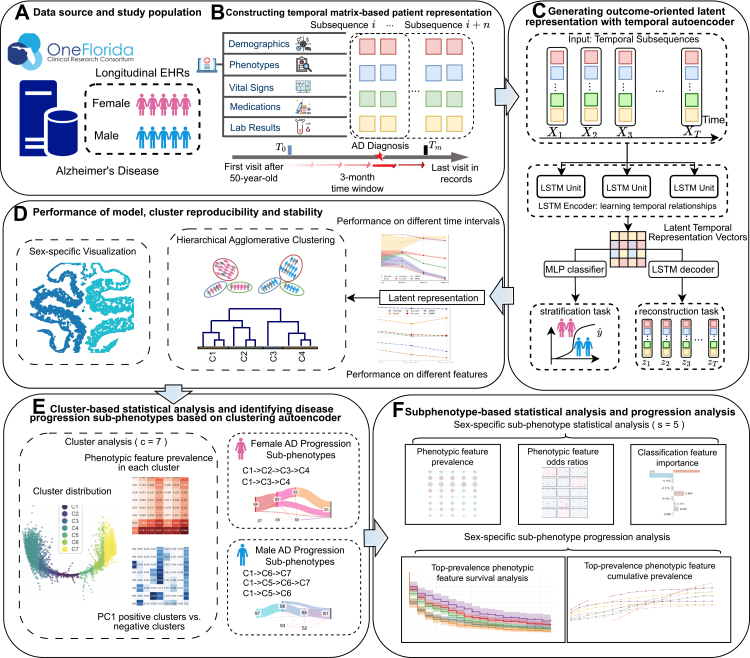
**The overview of the study. A**. Data source and cohort identification. **B**. The construction of longitudinal EHRs for individuals with AD. **C**. Sex-specific AD latent representation generation with our proposed autoencoder framework. **D**. Model optimisation and evaluation. **E**. Sex-specific AD progression sub-phenotypes identification and characterisation. **F**. Sex-specific AD progression sub-phenotypes analysis and interpretation.

In this study, we developed an autoencoder architecture to identify sex-specific AD progression sub-phenotypes using large-scale longitudinal EHRs from the OneFlorida+ clinical research network. Unlike traditional approaches that analyse associations between clinical characteristics and patient outcomes, our proposed framework can capture dynamic disease progression while accounting for sex stratification. Specifically, we first identified and collected AD cohorts with longitudinal EHRs from the OneFlorida+, constructing patient-specific temporal matrices to represent sequential clinical records. An autoencoder was developed involving a three-layer encoder and a three-layer decoder to generate sex-based latent representations that reflect disease trajectory. Using hierarchical agglomerative clustering methods, we grouped latent embeddings into distinct clusters representing different disease states. We further analysed and compared the distributions of phenotypic features across clusters and identified sex-specific AD sub-phenotypes based on major state transitions within patients’ progression pathways. These sex-specific sub-phenotypes were interpreted through phenotype-based statistical analysis and progression modelling. This framework provides insights into the heterogeneity of AD progression in terms of sex differences, aiding in personalised AD care. The overall workflow of this framework is presented in Fig. 1.

## Methods

### Data source and cohort identification

The AD cohort for this study was derived from the OneFlorida+ Clinical Research Consortium, covering more than 26 million participants across several states in the United States,^35^, ^36^, ^37^ which has been utilised in several recent studies for AD risk prediction and characterising disease progression trajectories.^29^^,^^38^^,^^39^ OneFlorida+ consists of diverse de-identified EHR-based information adhering to the PCORnet Common Data Model (CDM), including demographics, encounters, procedures, diagnoses, vital signs, lab results, and others. This dataset included individuals who met the extraction criteria, including: (1) age ≥50 years at the first visit; (2) at least two distinct encounters; (3) at least one diagnosis of AD, i.e., International Classification of Diseases, Ninth Revision, Clinical Modification (ICD-9-CM) codes: 331.0 and International Classification of Diseases, Tenth Revision, Clinical Modification (ICD-10-CM) codes G30.0, G30.1, G30.8, and G30.9^29^; (4) visit dates between January 2012 and July 2024); (5) prescribed with least of the antidementia medications (i.e., Donepezil, Memantine, Rivastigmine, or Galantamine); (6) at least one visit record ≥ three years before first AD diagnosis and one visit record ≥ one year after first AD diagnosis.

### Variable selection and preprocessing

We included demographic, diagnosis, medication, and laboratory variables and applied standardised preprocessing procedures prior to model development. For demographic variables, sex and race were encoded as binary features, and age was grouped into 10-year intervals. For longitudinal clinical events, including diagnoses and medications, codes were first mapped to standardised coding systems and then transformed into binary features within predefined time windows. Specifically, ICD diagnosis codes were mapped to Phecodes, an EHR-specific codebase that supports phenome-wide association studies (PheWAS).^40^ Medication codes from National Drug Codes (NDC) and RxNorm were mapped to Anatomical Therapeutic Chemical (ATC) classification codes.^41^ Lab measurements were represented using Logical Observation Identifiers Names and Codes (LOINC). Lab variables with more than 30% missing values were excluded, and the remaining values were imputed using the median. All these variables were one-hot encoded, and finally, we concatenated them into a patient-centred embedding, excluding sex, which was used as the prediction label.

### Constructing temporal patient representation using longitudinal EHRs

To construct patients' longitudinal progression trajectory, we first aggregated multiple measurements or events recorded at irregular time points and converted them into fixed time-interval blocks (e.g., 3-month time intervals) to form subsequences for each patient. Each block formed a vector representing a specific event type (e.g., diagnoses, medications, etc.). To model the progression patterns, we split each patient into multiple temporal sub-sequences. Fig. 1B and Supplementary Figure S1 present the construction of the AD temporal trajectory using longitudinal EHRs. For example, a diagnosis vector contained distinct diagnosis codes and their frequencies within a 3-month window. For the invariant variables like demographics (e.g., race), we treated them as static features and passed them at each time window. To define the longitudinal observation window for each patient, we selected the earliest encounter date within three years prior to AD onset as the start date ($T_{0}$), the first recorded AD diagnosis as the diagnosis date ($T_{diag}$), and the latest encounter after AD onset as the end date ($T_{m}$). Given N patients, the total number of time windows T, and the dimension of patient feature matrix D, we constructed the temporal patient representation. The representation is a matrix in which patients can be represented as a 3-tuple symbol and a sequence of vectors for patient i at visit $t_{j}$, where $t_{j}$ ∈ $\left\{t_{1\;},t_{2},\ldots ,t_{T}\right\}$ and each visit is denoted as $x_{i,t_{j}}\in R^{T\;\times \;D}$. To increase the quantity and augment the data, the original data (where each patient corresponds to a temporal matrix) has been divided into multiple subsequences. Starting from the start date $t_{1\;}$ (the date of the first visit), every 2 windows (e.g., 6 months) form one subsequence, until reaching the maximum length of the patient's EHR data. We then divided each patient into multiple subsequences with varying time lengths, starting from the index date, and new subsequences are created with 3-month increments (i.e., 6-month, 9-month, etc.) until the last encounter of patients within the end date. Each subsequence was treated as an independent temporal matrix sample and stacked into a three-dimensional model input. We also extracted the last subsequence from each patient to assess the influence of varying numbers of subsequences and their overlap. Therefore, the l-th subsequence can be represented as $x_{i,t_{j}}^{\left(l_{i}\right)}$, where $\left\{l_{1\;},l_{2},\ldots ,l_{N}\right\}$ is the index of each patient's subsequence. Finally, we obtained the entire temporal matrix with dimensions from (N,T,D) to $\left(\sum_{n\;=\;1}^{N}l_{n},T,D\right)$. We concatenated these subsequences in different time lengths to construct the AD progression trajectory for progression modelling.

### Sex-guided representation learning of individuals with AD using autoencoder

To extract latent embeddings of the subsequences of patients with AD, we first constructed a deep learning-based autoencoder, comprising a three-layer fully connected encoder and a symmetric decoder. We compared different models, including Multilayer Perceptron (MLP),^42^ Long Short-term Memory (LSTM), Gated Recurrent Unit (GRU),^43^ and Transformer.^44^ The encoder reduced the input feature dimensions to a 16-dimensional latent representation, while the decoder reconstructed the original input sequence. Furthermore, the learnt latent representation was fed into an MLP to classify patients by sex with learnt representation (Fig. 1C). Because the model jointly optimised reconstruction and sex classification tasks, the resulting latent representations reflected sex-associated disease patterns rather than purely unsupervised structures. The model was trained jointly using the Adam optimiser, summing the mean squared error (MSE) for reconstruction and binary cross-entropy (BCELoss) for classification. We utilised the area under the receiver operating characteristic curve (AUROC) to evaluate classification performance. We split the study cohort at the patient level into training (70%), validation (10%), and testing (20%) sets, and all subsequences derived from a given patient were assigned to the same set. We further performed an ablation study to evaluate the impact of subsequence length and selection of phenotype categories on model performance. Specifically, we tested various time intervals (i.e., 3-, 6-, 9-, and 12-month) for constructing patient subsequences, each containing longitudinal clinical records within the specified window. To assess the influence of input features, we examined different combinations of phenotype categories: (1) demographics and diagnoses, (2) demographics and medication, and (3) demographics and lab results.

### Clustering sex-specific latent representation of patients with AD

We further utilised different clustering algorithms (i.e., k-means,^45^ hierarchical agglomerative clustering,^46^ spectral clustering,^47^ and Gaussian mixture models [GMM]^48^) on the sex-specific subsequence representations of patients with AD, aiming at identifying the clusters (i.e., disease states). Each cluster represents a group of subsequences with similar latent representations derived from the autoencoder rather than predefined time periods. To determine the optimal clustering approach for identifying distinct disease states, we evaluated the reproducibility and stability of these methods. Regarding reproducibility, we employed the silhouette score,^49^ which measures how similar a data point is to its own cluster compared to other clusters. In parallel, we used the Adjusted Rand Index (ARI) and Adjusted Mutual Information (AMI), widely used metrics that quantify similarity between two clusters,^50^ where higher ARI or AMI indicates greater consistency across clustering iterations with stronger stability. We also evaluated the stability of clustering by the Dunn index and Davies-Bouldin index.^51^^,^^52^ To select the optimal number of clusters, the elbow method was used to identify a point where the rate of decrease in within-cluster variance slows, indicating a good trade-off between model complexity and cluster resolution. Further, we used principal component analysis (PCA) and t-SNE^53^ dimensionality reduction to visualise the distribution of the latent embeddings of patient sub-sequences. To quantitatively evaluate the relationship between clusters and the PCA components, we employed the Mann–Whitney U test,^54^ a non-parametric test used to compare two independent samples and assess whether they originate from the same distribution. In this context, the Mann–Whitney U test takes the PCA component from states as parameters, compares their ranks to assess whether they come from the same distribution, and returns the test statistic and p-value to indicate statistical significance. All analyses are conducted at a significance level of p-value < 0.05.^55^

### Deriving sex-specific AD progression sub-phenotypes

To identify sex-specific AD progression sub-phenotypes, we first extracted the temporal representations of each subsequence from the encoder's outputs. We then used the clustering algorithm to identify them into different clusters that exhibit similar temporal properties. Once the clusters of subsequences were identified, each patient's state at a given time point was determined by the cluster assignment of the corresponding subsequence. For each patient, the identified states were concatenated chronologically along their disease progression pathways, and persistent states were merged to retain the key transitions that capture the disease progression from one state to another. For instance, we suppose that a patient has four sub-sequences containing clinical information with different time lengths (e.g., 3, 6, 9, and 12 months). Each subsequence can be grouped into a cluster (i.e., state) denoted as $C_{i}\;\left(i=1,2,3,\ldots \right)$, assuming 3-month to $C_{1}$, 6-month to $C_{2}$, 9-month to $C_{3}$ , and 12-month to $C_{4}$. The progression trajectory of this patient would be represented as “$C_{1}\to C_{2}\to C_{3}\to C_{4}$”, which would be categorised as one of the sex-specific AD progression sub-phenotypes $S_{i}$. Subsequently, all patients with AD with the same progression pathway were categorised into one sub-phenotype. To visualise the composition and state transitions of each sub-phenotype, we calculated the distribution using Sankey diagrams. Specifically, we quantified the number of female and male patients within each sub-phenotype and mapped the proportion of distinct disease states along their respective progression pathways. For downstream analyses, we focused only on the major sub-phenotypes that included at least 100 patients with AD and ensured that each state within the trajectory contributed more than 1% of the total sub-phenotype population.

### Characterisation and interpretation of identified sub-phenotypes

To validate the co-occurrence between sex groups and sub-phenotype groups, we computed the Pearson correlation coefficients between sex and sub-phenotypes.^56^ For each phenotype $p_{k}$, we calculated its prevalence within sub-phenotype as the proportion of patients exhibiting $p_{k}$ at their latest encounter after AD onset $T_{m}$. We assessed phenotypic differentiation across sub-phenotypes using pairwise chi-square tests,^57^ with the Benjamini–Hochberg false discovery rate (BH-FDR) as the primary multiple-comparison correction.^58^ Bonferroni correction was additionally applied as a sensitivity analysis.^59^ The average statistical significance level (p-value) and the pooled odds ratio were from the chi-square tests and Fisher's exact tests comparing phenotype $p_{k}$ between $S_{i}$ and other sub-phenotypes. Moreover, to evaluate the discriminative power of $S_{i}$ and to identify key phenotypic predictors, we performed binary logistic regression models to classify female-dominant and male-dominant sub-phenotypes. Feature importance was determined by averaging the regression coefficients across runs. We enhanced clinical interpretability by mapping all phenotypes to Phecode Chapters,^60^ which classify phenotypes into 18 organ system categories. For visualisation and downstream analyses, we further restricted our focus to the phenotypes with the highest prevalence and statistically significant differences across all sub-phenotypes (i.e., p-value <0.05).

### Progression patterns of sub-phenotypes

To gain a deeper understanding of disease progression dynamics across sub-phenotypes, we performed survival analysis. The objective was to examine whether the presence of specific comorbidities within each sub-phenotype was associated with differences in risk and time to AD onset. We generated Kaplan–Meier survival curves^61^ across sub-phenotypes with specific comorbidity. Survival time was defined as the time from the index visit to the first recorded AD diagnosis. The proportional hazards assumption for the Cox models was assessed using Schoenfeld residual tests.^62^ To statistically compare survival probability differences between sub-phenotypes, we applied the two-sided log-rank test.^63^ We further employed the Cox proportional hazards model to estimate the relative risk of AD onset between female- and male-dominant sub-phenotypes. The Cox models were adjusted for potential confounders, including age, race, ethnicity, and number of encounters. Hazard ratios (HRs) and their corresponding 95% confidence intervals (CIs) were calculated using robust standard errors^64^ to ensure reliable inference. Additionally, we calculated the cumulative prevalence of phenotypes to capture comorbidity accumulation patterns. Prior studies^65^, ^66^, ^67^ have shown that rising prevalence of certain comorbidities may be associated with the onset of AD. Together, these analyses provide insights into the temporal dynamics and risk profiles of sex-specific AD progression sub-phenotypes and help characterise potential time windows for earlier monitoring based on comorbidity patterns.

### Ethics

This study complied with all relevant ethical regulations and was approved by the University of Florida Institutional Review Board (protocol no. IRB202202820). All patients’ clinical data were de-identified, and the institution waived the requirement for written informed consent. This study used no human biological samples and was conducted in accordance with the principles of the 2024 Declaration of Helsinki.

### Statistics

Continuous variables were summarised as mean (standard deviation), and categorical variables as counts and percentages. Group comparisons used t-tests, Wilcoxon rank-sum tests, and chi-square tests, as appropriate. Phenotypic comparisons used chi-square or Fisher's exact tests, with BH-FDR and Bonferroni corrections applied for multiple comparisons. Survival analyses used Kaplan–Meier curves, log-rank tests, and Cox proportional hazards models. Statistical significance was defined as p < 0.05.

### Role of funders

The funders had no involvement in the study design, data collection, analysis, interpretation, or manuscript writing.

## Results

### Cohort summary and characteristics

As shown in Table 1, we identified 1665 individuals with AD from the OneFlorida+ Clinical Research Network, consisting of 961 females (mean age at AD diagnosis: 77.1 ± 9.5 years) and 704 males (mean age at AD diagnosis: 76.1 ± 8.8 years). Cohort selection and sample size changes are shown in Supplementary Figure S2. According to the table, we found that females made up a slightly larger proportion than males (58% versus 42%) and were diagnosed at an older average age (77.1 years versus 76.1 years, p = 0.05). They had a longer mean follow-up duration than males (8.21 years vs 7.95 years, p = 0.002). Meanwhile, the average years before diagnosis were nearly the same between females and males (5.59 ± 1.73 years vs 5.6 ± 1.7 years, p = 0.82), whereas females had a longer post-diagnosis duration than males (2.62 ± 1.59 years vs 2.36 ± 1.48 years, p < 0.001). Regarding mortality rates, males showed a higher mortality rate (14%) compared to females (11%). We also observed racial and ethnic disparities, including a higher proportion of Hispanic individuals among females (26%) than males (25%), and a greater percentage of Black or African American individuals in females (16%) compared to males (15%). Conversely, a larger proportion of white individuals was found among males (79%) relative to females (77%). Two-sample t-tests, Wilcoxon rank-sum tests, and chi-square tests were used for comparisons between females and males. More details of other demographic and phenotypic characteristics of the cohort can be found in Table 1, Supplementary Tables S1 and S2.

**Table 1:** Descriptive statistics on the characteristics of the study cohort.

Characteristics	Total individuals with AD (N = 1665)	Females with AD (N = 961)	Males with AD (N = 704)	P-value
Demographics				
Age at AD diagnosis, mean (std)	76.7 (9.2)	77.1 (9.5)	76.1 (8.8)	0.05^a^
Female, N (%)	961 (58%)	961 (100%)	0 (0%)	b
Male, N (%)	704 (42%)	0 (0%)	704 (100%)	
Disease development, mean years (std)				
Follow-up durations	8.1 (1.70)	8.21 (1.68)	7.95 (1.72)	0.002^c^
Years before diagnosis	5.6 (1.72)	5.59 (1.73)	5.6 (1.7)	0.82^c^
Years after diagnosis	2.5 (1.55)	2.62 (1.59)	2.36 (1.48)	<0.001^c^
Mortality rate, N (%)	209 (13%)	109 (11%)	100 (14%)	0.037^d^
Hispanic, N (%)				0.71^d^
Hispanic	426 (26%)	248 (26%)	178 (25%)	
Not Hispanic	1230 (73%)	707 (73%)	523 (74%)	
No Hispanic information	9 (1%)	6 (1%)	3 (1%)	
Race, N (%)				0.55^d^
American Indian or Alaska Native	0 (0%)	0 (0%)	0 (0%)	
Asian	10 (1%)	5 (1%)	5 (1%)	
Black or African American	259 (16%)	157 (16%)	102 (15%)	
White	1298 (77%)	741 (77%)	557 (79%)	
Multiple race	15 (1%)	6 (1%)	9 (1%)	
Unknown	83 (5%)	52 (5%)	31 (4%)	

a Two-sample t-test.

b Compared group with no p-value computed.

c Wilcoxon Rank Sum Test.

d Chi-square test.

### Model performance for sex-stratified classification of individuals with AD

**Fig. 2:**
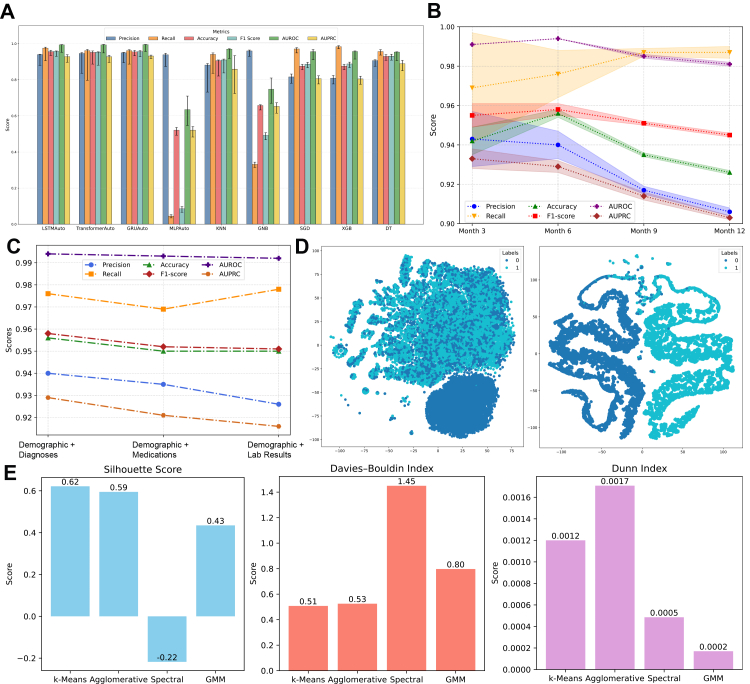
**Comparative performance of sex-stratified classification of patients with AD and clustering. (A)** The performance of sex-stratified AD classification with our proposed framework and other baselines. **(B)** The performance of the LSTM Autoencoder across different time points (Month 3, 6, 9, and 12) under different metrics. The plot shows the mean ± standard deviation (shaded areas), where larger shaded areas indicate higher variability (standard deviation) across measurements. **(C)** The impact of different EHR categories on the performance of the LSTM Autoencoder under different metrics. The model was evaluated using three feature sets: Demographic + Diagnoses, Demographic + Medications, and Demographic + Lab Results. **(D)** The comparison of t-SNE visualisations of original (left) and learnt (right) embeddings for patients with AD. Each point represents a patient with AD, coloured by sex label (“0” = male, “1” = female). **(E)** The evaluation of different clustering algorithms (k-Means, Agglomerative, Spectral, and GMM) applied to sex-stratified subsequences of patients with AD. Clustering performance is assessed using three internal validation metrics: Silhouette Score (higher is better), Davies–Bouldin Index (lower is better), and Dunn Index (higher is better), reflecting cluster compactness and separation.

Compared with the best-performing baseline model (Decision Tree, AUROC: 0.954; 95% CI: 0.906–0.955), autoencoder-based models demonstrated overall superior performance in identifying sex-stratified patients with AD. Among them, GRUAuto achieved the highest AUROC of 0.995 (95% CI: 0.957–0.996), followed closely by LSTMAuto and TransformerAuto (both 0.994). We evaluated subsequences across different temporal intervals and found that the 6-month interval achieved the best overall performance (accuracy: 0.956, F1: 0.958, AUROC: 0.994), while shorter and longer intervals showed trade-offs in precision and recall (Fig. 2B). We further compared different combinations of longitudinal EHR data types. Fig. 2C shows that the combination of demographics and diagnoses information achieved the highest AUROC (0.994), slightly surpassing demographics with medications (AUROC = 0.993) and demographics with lab results (AUROC = 0.992). Furthermore, we visualised the learnt representations using t-SNE and observed improved separation between male and female patient patterns after model training (Fig. 2D). We compared clustering performance across methods (Fig. 2E). K-means achieved the best silhouette score (0.62) and Davies–Bouldin index (0.51), while agglomerative clustering showed competitive performance with a high silhouette score (0.59) and the best Dunn index.

### Clusters of sex-stratified temporal subsequences from patients with AD

#### The selection of cluster number and visualisation

**Fig. 3:**
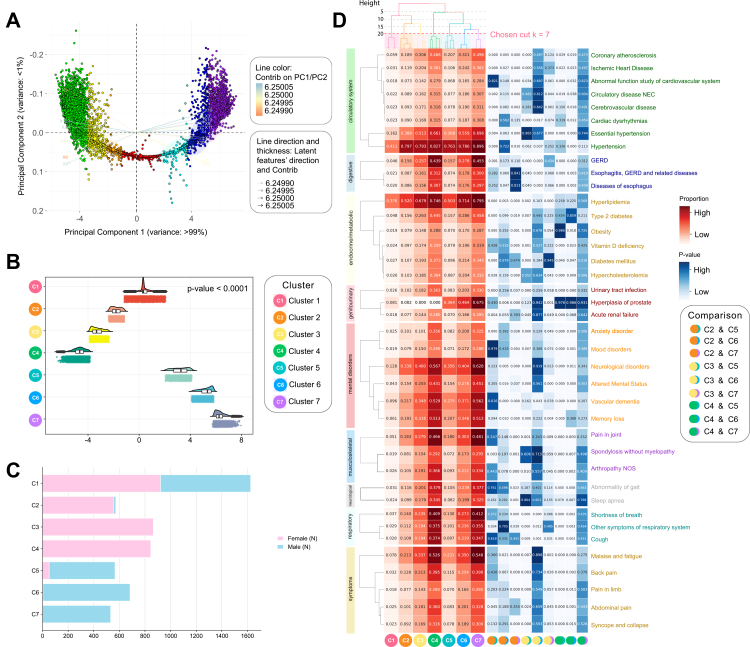
**The characterisation and analyses of derived clusters of sex-specific subsequences of patients with AD. (A)** The visualisation of seven identified clusters (C1–C7, henceforth termed as states) using PCA. Clusters C1–C7 represent disease states derived from clustering latent representations of temporal subsequences, rather than predefined temporal stages of AD progression. **(B)** Violin plot for cluster distribution on the first principal component (The first principal component explained >99% of the variance). **(C)** The number of female and male individuals with AD who had at least one subsequence assigned to each cluster (i.e., state). **(D)** The proportions of phenotypes in each state (left) and pairwise phenotypic comparisons between states (right). In the left panel, the intensity of the red colour indicates the proportion of each phenotype within a state, while the accompanying dendrograms depict the hierarchical relationships among states and phenotypes. In the right panel, the depth of the blue colour reflects the p-values from nine pairwise two-sided chi-square tests, with a significance threshold set at p < 0.05, and 0.000 indicates p < 0.0001.

To determine the optimal clusters (i.e., disease states) of sex-stratified patients with AD, we applied hierarchical agglomerative clustering to latent representations and tested cluster numbers from 2 to 10 using ARI (>0.8) and silhouette score (>0.5). The optimal clustering was obtained at a dendrogram height of 20, resulting in seven distinct clusters, denoted as C1–C7. Fig. 3A shows a PCA biplot with clear separation among the identified clusters, with PC1 accounting for over 99% of the variance and exhibiting significant variation across the seven clusters (Fig. 3A). To statistically validate the differences in PC1 distributions among clusters, we conducted a two-sided Mann–Whitney U-test (p-value <0.0001; Fig. 3B). Cluster C4 was positioned at the most negative end along PC1 (−4.96 ± 0.57), while Cluster C7 was positioned at the most positive end (6.23 ± 0.64). According to Fig. 3C, we also found that the sex distribution differs across clusters. For example, Cluster C1 displayed a relatively balanced distribution (921 females and 704 males with AD). In contrast, Clusters C2, C3, and C4 mainly consisted of females (percentage = 58%, 90%, and 88%), whereas Clusters C5, C6, and C7 were male-dominant, comprising 73%, 97%, and 75% of males with AD, respectively. These patterns suggest distinct sex-specific trajectories in AD progression.

#### Phenotypic characteristics of distinct clusters

The phenotype proportions within each cluster are displayed in Fig. 3D (left) and detailed in Supplementary Data 1, highlighting the phenotypic variability and heterogeneity across disease states. In C1, we can see that the most prevalent conditions included hypertension (41%, circulatory system), hyperlipidaemia (38%, endocrine/metabolic), essential hypertension (16%, circulatory system), and neurological disorders (13%, mental disorders). The results revealed that C4 and C7 exhibited a notable prevalence in multiple phenotypes. For example, hypertension was observed in 83% and 90% of patients in C4 and C7, respectively, and hyperlipidaemia in 75% and 80%. In contrast, states C1 and C5 exhibit comparatively lower comorbidity burdens, with hypertension occurring in only 41% and 76% of patients in C1 and C5, respectively. These findings suggested that C4 and C7 represent disease states characterised by a high burden of comorbidities, while C1 and C5 correspond to disease states with relatively fewer comorbid conditions among patients with AD. Additional analysis (Supplementary Table S3) revealed that state C4 had the longest mean follow-up duration (8.32 years), whereas state C5 had the shortest mean follow-up duration (7.98 years). Across all clusters, circulatory and endocrine/metabolic conditions, particularly hypertension, essential hypertension, and hyperlipidaemia, emerged as the top 3 most prevalent comorbidities across AD disease states.

#### Analysis of phenotypic difference in pairwise clusters

To further assess phenotypic differences between states, we performed pairwise two-sided chi-square tests with the BH-FDR and Bonferroni correction. The results (Fig. 3D, right) indicated significant differences regarding the top three prevalent phenotypes (i.e., hypertension, hyperlipidaemia, and essential hypertension) in several cluster pairs, including C2 & C5 (p-value <0.001, p-value <0.001, p-value = 0.004) and C2 & C7 (p-value = 0.01, <0.001, <0.001). Other pairs, such as C3 & C5, C4 & C5, and C4 & C6, also showed significant differences in multiple phenotypes like coronary atherosclerosis and gastro-oesophageal reflux disease (GERD), suggesting distinct comorbidity profiles across states, which is crucial to better understand the sex difference in AD progression. In contrast, clusters C4 and C7 displayed highly similar phenotypic distributions, with no significant differences across broader categories such as endocrine/metabolic, genitourinary, musculoskeletal, and symptom-related conditions. Moreover, we also found no significant differences in C3 & C6 and C4 & C7 pairs across broader phenotypic categories.

### Sex-specific AD progression sub-phenotype identification

**Fig. 4:**
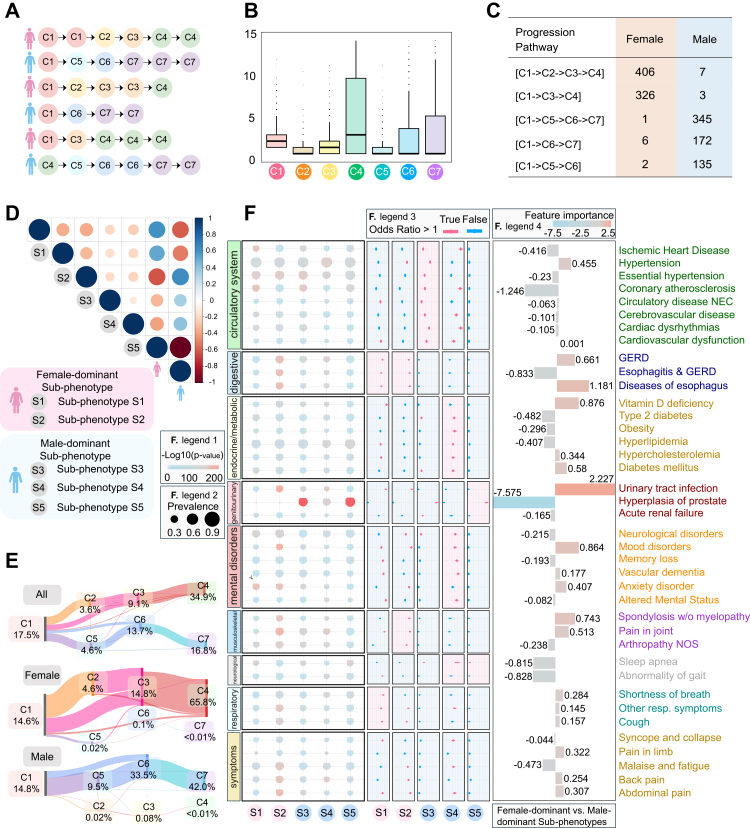
**The derivation and characterisation of sex-specific AD progression sub-phenotypes. (A)** The examples of the construction of patients' progression pathways. **(B)** The frequency of each disease state appeared within individual patient progression pathways. **(C)** The number and progression pathways of the five primary identified sex-specific AD sub-phenotypes. **(D)** The correlation between AD sub-phenotype and sex. **(E)** The Sankey diagrams of AD progression pathways for all patients (top), females with AD (middle), and males with AD (bottom). **(F)** The phenotype-based analyses of the five sex-specific AD sub-phenotypes: prevalence, statistical significance, and predictive importance. Sub-phenotypes S1–S5 represent patient-level AD progression trajectories defined by common sequences of disease states (C1–C7) observed across patients. S1, S2 are female-dominant sub-phenotypes, and S3–S5 are male-dominant sub-phenotypes. The bubble maps (left) display the proportions and statistical significance of phenotypes within each sub-phenotype, where bubble size corresponds to -log_10_ (p-value). The forest plot (middle) presents the odds ratios (ORs) of phenotype proportions across sub-phenotypes, with red markers indicating ORs greater than 1. The red background highlights phenotypes particularly associated with their respective sub-phenotypes. The pyramid plot (right) illustrates feature importance for classifying female- and male-dominant sub-phenotypes, with bar length representing the magnitude and direction of each phenotype's contribution.

Fig. 4A illustrates the example of sex-specific AD sub-phenotypes with distinct progression pathways, represented by temporal clusters within the same patient along the timeline. We further quantified how frequently each disease state occurred within individual patient trajectories (Fig. 4B). The results suggest that Clusters C4 and C7 appeared most frequently and were sustained for longer durations compared to other clusters. We then consolidated consecutive identical states (e.g., C1→C6→C7→C7 to C1→C6→C7) to generate a refined progression pathway for each patient with AD. Subsequently, we counted the number of patients sharing the same refined pathways and identified five primary sex-specific progression patterns (Fig. 4C) and their trajectories (Fig. 4E). These sub-phenotypes, denoted as S1–S5, represent distinct progression pathways, where (1) S1: C1→C2→C3→C4, (2) S2: C1→C3→C4, (3) S3: C1→C5→C6→C7, (4) S4: C1→C6→C7, and (5) S5: C1→C5→C6. According to Fig. 4C, we found that sub-phenotypes S1 and S2 were predominantly composed of females with AD, with 406 and 326 cases, but only 7 and 3 males, respectively. In contrast, S3, S4, and S5 are male-dominant AD sub-phenotypes, consisting of 345 vs 1, 172 vs 6, and 135 vs 2 for male and female cases. Additionally, correlation analysis further confirmed that females with AD showed a stronger correlation with S1 and S2, whereas males with AD were closely associated with sub-phenotypes S3, S4, and S5 (Fig. 4D). Interestingly, we noticed that in the flow, the states C2, C3, and C4 only existed in female-dominant sub-phenotypes S1 and S2, whereas C5, C6, and C7 were present in male-dominant sub-phenotypes S3–S5. The results further validated different progression patterns in females and males with AD with distinct sub-phenotypes.

### Phenotypic characteristics of sex-specific sub-phenotypes

We analysed the five AD sub-phenotypes (S1–S5) and found clear differences in demographics and clinical features, summarised in Table 2. We observed that S1 had the oldest age at AD diagnosis (mean age: 77.2 ± 9.8 years), the longest follow-up duration (8.31 years), and the longest disease duration (2.64 years). In contrast, S5 exhibited the youngest mean age at AD diagnosis (mean age: 74.8 ± 9.5 years), along with the shortest follow-up (7.39 years) and disease duration (2.14 years). Racial composition varied across groups, with S5 involving the highest proportion of Hispanic patients (n = 38, 28%), S2 showing more Black or African American patients (n = 60, 18%), and S3 having the highest proportion of White patients (n = 279, 80%). In terms of the three most common comorbidities of AD, neurological disorders were most prevalent in S3 (41%), followed by S4 (39%) and S1 (36%). Meanwhile, cardiovascular diseases also showed the highest prevalence in S3 (23%), followed by S2 and S4 (20% and 19%, respectively). Diabetes-related conditions were most prevalent among patients with AD in sub-phenotype S4 (52%). More demographic and phenotypic characteristics for each sub-phenotype are provided in Table 2 and Supplementary Table S4. To evaluate the robustness of these identified clusters and sub-phenotypes, we assessed their stability by varying key hyperparameters. The clusters and sub-phenotypes remained highly consistent across different settings, as evidenced by high ARI and AMI values (Supplementary Tables S5–S7). Additionally, we evaluated the impact of sex supervision on model performance, with details and results shown in Supplementary Figure S3 and Supplementary Table S8. We also performed a sensitivity analysis using only the last subsequence from each patient (Supplementary Figure S4).

**Table 2:** Descriptive statistics of five identified primary sex-specific AD sub-phenotypes.

Characteristics	S1 (N = 413)	S2 (N = 329)	S3 (N = 346)	S4 (N = 178)	S5 (N = 137)
Demographics					
Age at AD diagnosis, mean (std)	77.2 (9.8)	76.9 (9.2)	76.2 (8.7)	77.1 (8.3)	74.8 (9.5)
Female, N (%)	406 (98%)	326 (99%)	1 (0%)	6 (3%)	2 (2%)
Male, N (%)	7 (2%)	3 (1%)	345 (100%)	172 (97%)	135 (98%)
Disease development, mean years (std)					
Follow-up durations	8.31 (1.61)	8.29 (1.7)	8.24 (1.57)	8.05 (1.67)	7.39 (1.80)
Years before diagnosis	5.67 (1.73)	5.69 (1.76)	5.76 (1.66)	5.68 (1.80)	5.25 (1.52)
Years after diagnosis	2.64 (1.56)	2.6 (1.54)	2.48 (1.46)	2.37 (1.52)	2.14 (1.42)
Hispanic, N (%)					
Hispanic	111 (27%)	84 (26%)	85 (25%)	43 (24%)	38 (28%)
Not Hispanic	300 (72%)	243 (73%)	260 (75%)	134 (75%)	98 (71%)
No Hispanic information	2 (1%)	2 (1%)	1 (0%)	1 (1%)	1 (1%)
Race, N (%)					
American Indian or Alaska Native	0 (0%)	0 (0%)	0 (0%)	0 (0%)	0 (0%)
Asian	3 (1%)	0 (0%)	3 (1%)	1 (1%)	1 (1%)
Black or African American	56 (14%)	60 (18%)	55 (16%)	26 (15%)	12 (9%)
White	328 (78%)	250 (76%)	279 (80%)	139 (77%)	110 (80%)
Multiple race	2 (1%)	2 (1%)	3 (1%)	2 (1%)	3 (2%)
Unknown	24 (6%)	17 (5%)	6 (2%)	10 (6%)	11 (8%)
Comorbidity, N (%)					
Neurological	149 (36%)	113 (34%)	143 (41%)	70 (39%)	37 (27%)
Cardiovascular	68 (16%)	65 (20%)	78 (23%)	34 (19%)	21 (15%)
Diabetes-related	171 (41%)	157 (48%)	178 (51%)	92 (52%)	50 (36%)

To characterise the phenotypic characteristics of sub-phenotypes, we investigated the prevalence of the top phenotypic features (Fig. 4F, left; Supplementary Data 2). Among these, sub-phenotype S3 had the largest proportion of hypertension at 80%, a condition also prevalent in other sub-phenotypes. Similarly, hyperlipidaemia and essential hypertension were frequently observed in all sub-phenotypes, aligning with the top three phenotypes previously identified in individual disease states. Among male-dominant sub-phenotypes, aside from the top three phenotypes, hyperplasia of prostate, as a male-specific phenotype, was also notably prevalent (64%). Additionally, S5 showed substantially higher proportions in most phenotypes, whereas S4 consistently exhibited lower proportions. In contrast, female-dominant sub-phenotype S2 showed elevated proportions of neurological disorders (50%) and malaise and fatigue (46%). We also examined phenotypic differences across sub-phenotypes (Fig. 4F). It is revealed that female-dominant sub-phenotypes showed elevated effect sizes in digestive disorders, such as GERD. Additionally, S1 was associated with increased effect sizes for respiratory symptoms, while S2 exhibited stronger associations with musculoskeletal disorders. In contrast, the male-dominant sub-phenotypes demonstrated higher effect sizes for neurological conditions, particularly sleep apnoea. S3 and S5 were also highly associated with genitourinary disorders (e.g., hyperplasia of the prostate), while S4 was enriched for endocrine/metabolic conditions (e.g., type 2 diabetes). To assess how well the sex-specific AD sub-phenotypes can be distinguished, we trained a model to classify female- and male-dominant sub-phenotype groups. Hyperplasia of prostate (genitourinary, coefficient = −7.575) was the most prominent predictor for the male-dominant group, while urinary tract infection (genitourinary, coefficient = 2.227) was strongly associated with the female-dominant group. Other top predictors for the male-dominant group included coronary atherosclerosis and abnormal gait, whereas oesophagus and mood disorders were important for the female-dominant group.

### Analysis of AD progression patterns in sex-specific sub-phenotypes

**Fig. 5:**
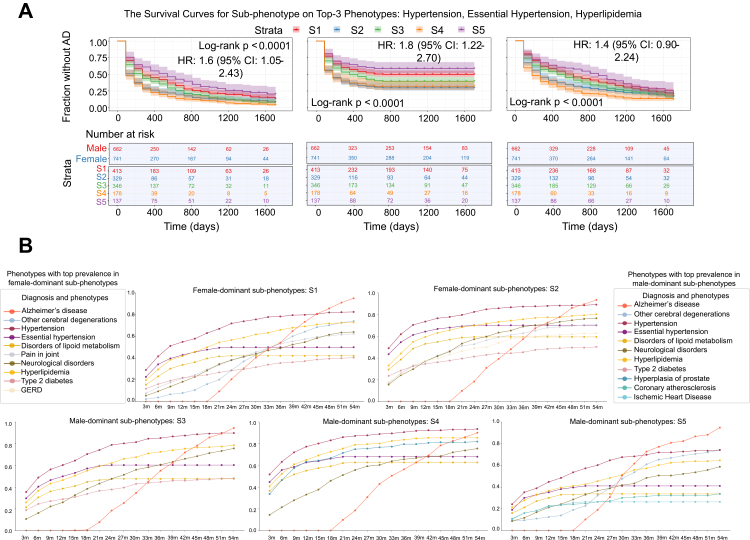
**Survival analysis and cumulative prevalence curves for top comorbid phenotypes associated with AD onset across five identified sex-specific sub-phenotypes. (A)** Kaplan–Meier survival curve depicting time to AD onset across the top three comorbid phenotypes (hypertension, essential hypertension, hyperlipidaemia) for each sub-phenotype. The curves represent the proportion of patients without AD over time, with the Log-rank test's p-values indicating significant differences between sub-phenotypes (p < 0.0001). The number of patients at risk is displayed for each sub-phenotype at different time points (unit: day). **(B)** Cumulative prevalence curves for the five sex-specific AD sub-phenotypes, showing the accumulation of high-prevalence comorbidities or AD onset over time. Each point represents the cumulative proportion of patients diagnosed with either AD or one of the most prevalent comorbid phenotypes, calculated at 3-month intervals.

In survival analysis (Fig. 5A), our findings revealed that the male-dominant sub-phenotype S4 had the fastest decline in survival probability without AD compared to other sub-phenotypes when either hypertension or hyperlipidaemia was exposed within patients. In contrast, female-dominant S2 had the fastest decline in survival probability for those who were under essential hypertension. Furthermore, S1 and S5 demonstrated the slowest decline in survival probability across all three conditions. These results, along with the two-sided log-rank tests (Log-rank p-value <0.0001), indicate significant differences in survival probabilities across sub-phenotypes under different comorbidities. Cox proportional hazards analysis further showed that both essential hypertension and hypertension were significantly associated with increased AD risk. The hazard ratios (HRs) under all three comorbidity conditions were greater than 1, but only essential hypertension and hypertension had confidence intervals that did not include 1 (hypertension: HR = 1.6 [95% CI: 1.05, 2.43]; essential hypertension: HR = 1.8 [95% CI: 1.22, 2.70]; hyperlipidaemia: HR = 1.4 [95% CI: 0.901, 2.243]). The adjusted results of Cox models are provided in Supplementary Table S9. We also calculated the cumulative prevalence of selected comorbidity phenotypes along with AD prevalence across different sub-phenotypes over time. As shown in Fig. 5B, AD prevalence across all sub-phenotypes began to increase around Month 18 and continued to rise until Month 54. At the sub-phenotype level, we found that S2 and S4 exhibited the earliest accumulation of comorbidities, with each exceeding 30% prevalence within the first 3 months. Notably, S5 showed AD prevalence exceeding essential hypertension as early as Month 27–30 and all top comorbidities in Month 33–36. Although S1 also showed a relatively rapid increase in AD prevalence, it maintained a slower decline in survival probability, while S3 followed a relatively stable progression trajectory.

## Discussion

In this work, we developed an autoencoder-based framework to identify sex-specific sub-phenotypes of AD progression using longitudinal EHRs and uncover their associated clinical characteristics. In this context, sex denotes biological attributes, specifically classifying individuals as male or female. By integrating a deep learning-based autoencoder with clustering, we generated temporal representations of patient trajectories and grouped them into different clusters (i.e., disease states), which were then combined to define progression sub-phenotypes. Our model demonstrated strong performance and stability, identifying five primary sex-specific AD sub-phenotypes with distinct clinical characteristics, highlighting the heterogeneous nature of AD progression. Our findings emphasise the importance of incorporating sex as a key factor in AD research. In our cohort, females showed a larger proportion of patients with AD, with an older age of diagnosis and a longer time of disease development on average. However, males with AD had a higher mortality rate (14%) compared to females (11%), consistent with findings from previous studies.^1^^,^^27^^,^^68^ Through our clustering analysis, we identified seven disease states (C1–C7), with C4 containing the largest number of patient subsequences. Across the identified disease states, circulatory and metabolic conditions were the most common comorbidities, with hypertension (41%) and hyperlipidaemia (38%) showing the highest prevalence. Although previous studies have established associations between these comorbidities and sex-specific AD groups, our study further revealed both similarities and differences in comorbidity patterns across disease states. For example, some states (e.g., C4 and C7) shared similar phenotype proportions across categories, such as endocrine/metabolic systems, while others (e.g., C2 and C5) showed significant differences in phenotypes like hypertension and hyperlipidaemia. Importantly, these states exhibited different sex distributions, indicating that sex may be associated with the progression trajectories observed throughout the disease course.

By aggregating disease states over time, we identified five major AD sub-phenotypes, including two female-dominant (S1 and S2), and three male-dominant (S3–S5), each exhibiting distinct progression pathways (Fig. 4C). Across all these identified AD sub-phenotypes, hypertension and hyperlipidaemia were the most prevalent comorbidities, consistent with previous evidence that vascular dysfunction^69^, ^70^, ^71^, ^72^ and neuroinflammation^73^ are related to AD progression. Among female-dominant subtypes, S1 had a lower overall comorbidity burden compared to S2 but was characterised by a higher prevalence of respiratory conditions, including shortness of breath, cough, and other respiratory symptoms. While respiratory diseases are not typically considered to be associated with AD, our findings suggest that they may co-occur with specific AD progression patterns among females. Previous studies suggest that factors such as disrupted sleep patterns,^74^ increased exposure to air pollution,^75^ or susceptibility to pulmonary infections,^76^ have been identified in elderly patients with AD. In contrast, S2 included a higher comorbidity burden and was enriched with neurological and mental disorders, consistent with known associations between AD and conditions such as depression in women.^77^ Among male-dominant sub-phenotypes, S3 had the largest number of males and was associated with circulatory and genitourinary conditions, while S4 exhibited a lower comorbidity burden and was mainly characterised by metabolic disorders such as diabetes and obesity. S5 showed the highest comorbidity burden, with prominent genitourinary disorders (e.g., hyperplasia of prostate) and neurological conditions (e.g., sleep apnoea and abnormality of gait). Additionally, some comorbidities, such as genitourinary disorders, were observed in both sexes but manifested differently. Notably, we also identified less-studied associations, such as digestive disorders (e.g., GERD and oesophagitis) in females, suggesting potential sex-specific pathways in AD progression that warrant further investigation.

We further examined disease progression patterns and observed that the identified sub-phenotypes exhibited distinct trajectories. For example, S4 (C1 → C6 → C7) lacks the intermediate state C5 compared with S3 (C1 → C5 → C6 → C7), suggesting a potentially more direct transition through advanced disease stages. Survival analysis showed that time to AD diagnosis differed across sub-phenotypes among patients with various comorbidities. The decline rates in survival probabilities prior to AD onset varied according to exposure to the three most prevalent comorbid conditions. Among patients with hypertension, the male-dominant sub-phenotype S4 had the shortest time to AD diagnosis, followed by S2, a female-dominant sub-phenotype, suggesting a potential association between greater comorbidity burden and accelerated disease onset. In contrast, S1 (female-dominant) and S5 (male-dominant) showed the slower decline of survival probabilities and were characterised by relatively lower comorbidity burden. In patients with essential hypertension, S2 had the shortest time to AD diagnosis, consistent with previous reports that postmenopausal hypertension and depressive symptoms may be related to dementia progression in women.^78^ These findings further showed that vascular and psychiatric comorbidities are relevant clinical features in sex-specific AD progression. Moreover, cumulative prevalence analysis confirmed that S5 had AD prevalence exceeding essential hypertension earlier, suggesting the need for early monitoring during the initial stages of comorbidity development. By contrast, S4 exhibited a comparatively slower accumulation of AD prevalence over time. Collectively, these underscore the importance of examining associations between comorbidities and AD progression in a sex-specific context, highlighting opportunities for early monitoring in high-risk subgroups.

While this study provides important insights into sex-specific AD sub-phenotypes using advanced AI methods, several limitations should be acknowledged. First, the reliance on EHR-recorded sex may not reflect gender identity and may not fully capture biological sex characteristics. Second, because sex information was incorporated into the representation learning process, some of the identified sex-specific sub-phenotypes may partially reflect the modelling objective rather than arising solely from unbiased clustering of longitudinal disease patterns. Third, as an observational study based on EHR data, our findings describe associations rather than causal relationships, and the clinical utility of the identified sub-phenotypes will require validation in prospective studies and clinical trials. Lastly, this study may preferentially include patients with more complete longitudinal and medication records and lacks detailed cognitive assessment (e.g., the Mini-Mental State Exam) and AD-specific biomarkers (e.g., neuroimaging and cerebrospinal fluid measures), which could provide additional insights into disease progression. Future research could focus on three key directions. First, validating the identified sub-phenotypes in larger and more diverse populations is critical to improve robustness and clinical applicability, potentially leveraging federated learning to integrate data across multiple institutions. Second, incorporating multimodal and multi-omics data, such as genomic, transcriptomic, and imaging data, will help uncover the biological mechanisms underlying sex differences in AD progression and generate hypotheses for future sex-specific therapeutic studies. Third, developing more advanced and interpretable computational models to track individual disease trajectories over time will enable more precise monitoring, early detection, and personalised intervention for patients with AD.

## Contributors

R.Y. conceived the idea. W.M., Q.Y., and R.Y. designed the experiments. W.M. and Q.Y. performed the experiments. R.Y. and J.X. developed the initial concept. W.M. wrote the initial manuscript with support from R.Y. Q.Y., J.X., Y.H., Q.S., C.W., A.M., Q.M., L.S., J.B., and R.Y. provided critical feedback and helped shape the research, analysis, and manuscript. R.Y. supervised the project. W.M. and R.Y. have fully accessed and verified the underlying data. All authors read and approved the final version of the manuscript.

## Data sharing statement

The OneFlorida+ Data Trust is accessible to investigators for research purposes through formal policies and procedures established by OneFlorida+ Executive Committee (OneFloridaOperations@health.ufl.edu). Researchers can initiate and complete Prep-to-Research Data Query via the OneFlorida+ Application Portal (https://webportalapp.com/sp/login/onefl_preptoresearch). OneFlorida+ Informatics for Integrating Biology and the Bedside (i2b2) (https://onefl.net/front-door/onefli2b2/) facilitates cohort discovery and conducts descriptive analyses, which provide aggregate patient counts, sociodemographic and health characteristics, and healthcare practice demographics. Access to individual-level data requires a formal data request submission and IRB approval. For information on data access procedures, investigators can schedule a consultation (https://onefl.net/front-door/consultation/). The codes for this study are publicly available at https://github.com/UF-HOBI-Yin-Lab/AD_sex_subtype. All hyperparameter settings are provided in Supplementary Table S10.

## Declaration of interests

The authors declare no competing interests.
