# Supplementary material for: Identifying sex-specific sub-phenotypes of Alzheimer's disease progression using longitudinal electronic health records
Source: eBioMedicine. 2026 Jul 16;130:106391. doi: 10.1016/j.ebiom.2026.106391 (PMC13383015; doi:10.1016/j.ebiom.2026.106391)
Supplement: Supplementary Information [file mmc3.docx]

**Supplementary Information**

**Identifying Sex-Specific Sub-phenotypes of Alzheimer's Disease Progression Using Longitudinal Electronic Health Records**

Weimin Meng^1^, Qiang Yang^1^, Jie Xu^1^, Yu Huang^2^, Cankun Wang^3^, Qianqian Song^1^, Lixin Song^4^, Jiang Bian^2^, Qin Ma^3^, Anjun Ma^3^, ^*^ Rui Yin^1, *^

^1^ Department of Health Outcomes & Biomedical Informatics, University of Florida, Gainesville, FL, 32611, USA

^2^ School of Medicine, Indiana University, Indianapolis, IN, 46202, USA

^3^ Department of Biomedical Informatics, Ohio State University, Columbus, OH, 43210, USA

^4^ School of Nursing, University of Texas Health Science Center at San Antonio, San Antonio, TX, 78229, USA

*Correspondence to: [ruiyin@ufl.edu](mailto:ruiyin@ufl.edu) (R.Y.), [anjun.ma@osumc.edu](mailto:anjun.ma@osumc.edu) (A. M.)

**Supplementary Figures**


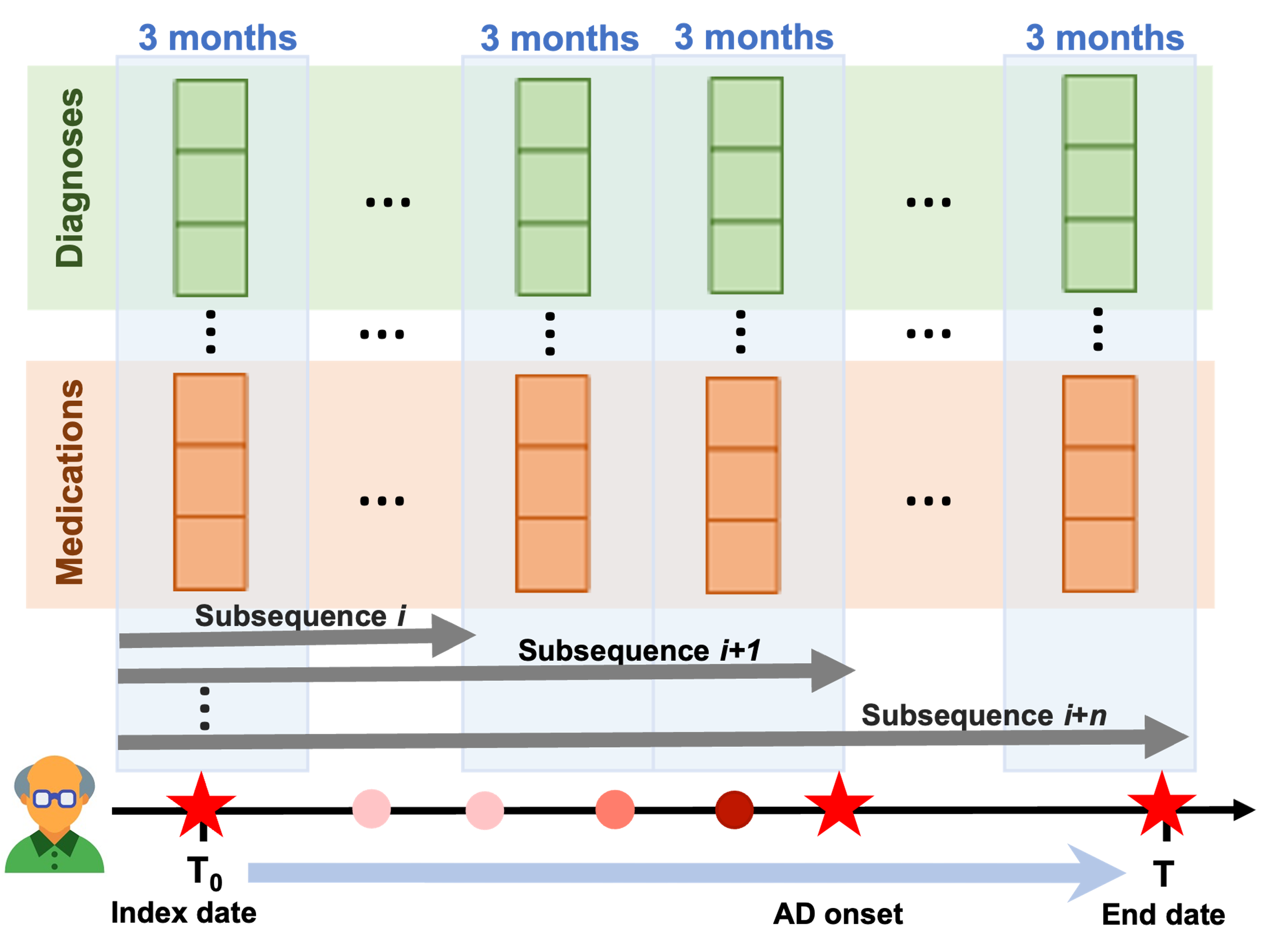


**Supplementary Figure 1. The construction of Alzheimer’s Disease (AD) temporal trajectory using EHRs.** Relevant EHR data for each patient was aggregated in 3-month blocks (i.e., time intervals) into a set of vectors (e.g., Diagnoses, Medications). A vector contains unique clinical variables and their frequencies within a time interval. For the invariant variables, demographic, we treated them as static features and passed them at each time interval. We then divided each patient’s vectors into multiple subsequences with varying time lengths, starting from the index date, and new subsequences are created with 3-month increments (i.e., 6-month, 9-month, etc.) until the last encounter of patients within the end date.


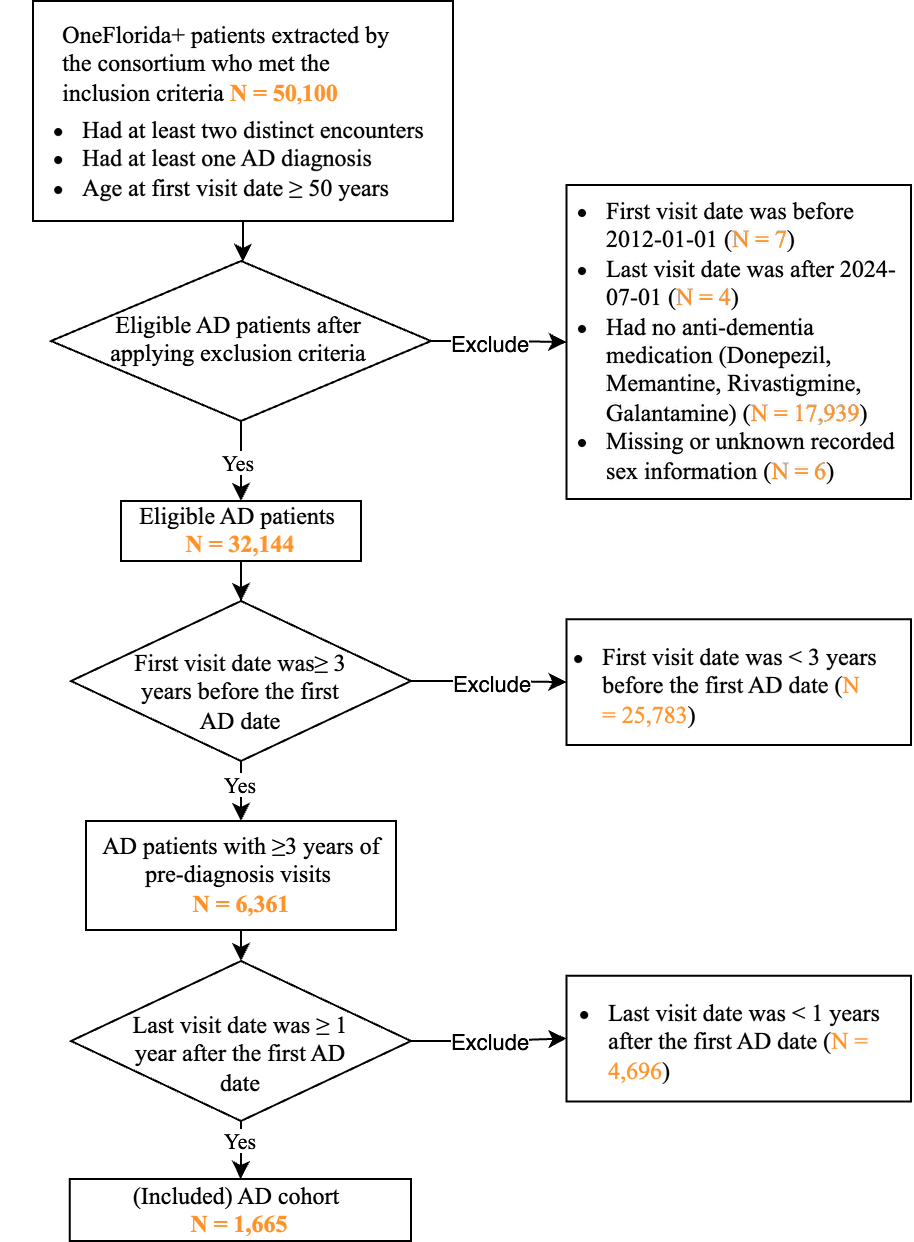


**Supplementary Figure 2. Cohort selection workflow for identifying the study population of patients with AD.** The initial de-identified dataset provided by the OneFlorida+ consortium included 50,100 patients who met the predefined extraction criteria (age ≥ 50 years at the first visit, at least two distinct encounters, and at least one AD diagnosis). From this candidate cohort, we applied several filtering steps to ensure clinical consistency and sufficient longitudinal observation. Patients were excluded if their visits fell outside the study observation window or if they had no anti-dementia medications. To enable trajectory modelling, we further required each patient to have at least three years of clinical records prior to the first AD diagnosis and at least one year of follow-up after diagnosis. After applying all inclusion and exclusion criteria, the final analytical cohort consisted of 1,665 patients with AD. The number of patients retained at each filtering step is shown in the workflow.

**Supplementary Figure 3. Unsupervised and sex-linked-feature exclusion sensitivity analyses for evaluating supervised sex-specific AD progression subphenotypes.** (A) Latent representations learned by an unsupervised autoencoder without the sex-classification task. Points are coloured by unsupervised subphenotype assignment and by EHR-recorded sex, showing limited sex separation in the purely unsupervised representation. (B) Overlap between sex-stratified unsupervised subphenotypes and supervised sex-specific subphenotypes. Separate unsupervised autoencoders were trained in female and male patients, and the resulting subphenotypes were compared with the supervised subphenotypes. (C) Comparison of sex-stratified unsupervised subphenotypes and supervised sex-specific subphenotypes. (D) Comparison of phenotype associations with sex and with AD progression clusters using chi-square tests and Cramér’s V. Phenotypes were ranked by association strength with sex and with clusters to assess whether cluster-associated features were reducible to sex-associated features. (E) Supervised representations after excluding the top 10%, 20%, and 30% sex-linked features, showing that the subphenotype structure remained stable after removal of strongly sex-linked features.

**Supplementary Figure 4. Robustness of clustering using a single final subsequence per patient.** PCA visualisation of latent representations generated from a sensitivity analysis using only the final subsequence from each patient. In this analysis, each patient contributed exactly one analytic unit, removing differential weighting due to variable numbers of subsequences per patient. Clusters are partially consistent with the originally identified clusters (ARI = 0.52, AMI = 0.68).

**Supplementary Tables**

**Supplementary Table 1.** Phecodes for the three most common comorbidities of AD.

| **Disease Category** | **Neurological disorders^1–3^** | **Cardiovascular diseases^4,5^** | **Diabetes^6,7^** |
| --- | --- | --- | --- |
| Phecodes | Phe_Memory loss,  Phe_Altered mental status,  Phe_Transient alteration of awareness,  Phe_Hallucinations,  Phe_Schizophrenia and other psychotic disorders,  Phe_Schizophrenia,  Phe_Paranoid disorders,  Phe_Mood disorders,  Phe_Bipolar,  Phe_Depression,  Phe_Major depressive disorder,  Phe_Suicidal ideation,  Phe_Anxiety disorders,  Phe_Anxiety disorder,  Phe_Phobia,  Phe_Personality disorders,  Phe_Eating disorder,  Phe_Anorexia nervosa,  Phe_Sleep disorders,  Phe_Insomnia. | Phe_Essential hypertension,  Phe_Hypertension,  Phe_Hypertensive heart and/or renal disease,  Phe_Other forms of chronic heart disease,  Phe_Ill-defined descriptions and complications of heart disease,  Phe_Hypertensive heart disease,  Phe_Congestive heart failure, nonhypertensive, Phe_Other chronic ischemic heart disease, unspecified,  Phe_Heart failure with reduced EF [Systolic or combined heart failure],  Phe_Acute rheumatic heart disease | Phe_Type 2 diabetes,  Phe_Diabetes mellitus,  Phe_Type 2 diabetes with renal manifestations,  Phe_Type 2 diabetes with neurological manifestations,  Phe_Polyneuropathy in diabetes,  Phe_Type 1 diabetes,  Phe_Type 2 diabetes with ophthalmic manifestations,  Phe_Diabetes type 2 with peripheral circulatory disorders,  Phe_Type 1 diabetes with neurological manifestations,  Phe_Diabetes type 1 with peripheral circulatory disorders,  Phe_Type 1 diabetes with ophthalmic manifestations,  Phe_Type 2 diabetes with ketoacidosis |

**Supplementary Table 2.** Descriptive statistics on the characteristics of the study cohort.

| **AD Cohort, N (%)** | **Total AD Patients**  **(N = 1,665)** | **Female AD Patients**  **(N = 961, 58%)** | **Male AD Patients**  **(N = 704, 42%)** |
| --- | --- | --- | --- |
| **Demographics** | | | |
| Age at AD diagnosis, mean (std) | 76.7 (9.2) | 77.1 (9.5) | 76.1 (8.8) |
| **Disease development, mean (std)** | | | |
| Follow-up durations | 8.1 (1.70) | 8.21 (1.68) | 7.95 (1.72) |
| Years before AD diagnosis | 5.6 (1.72) | 5.59 (1.73) | 5.6 (1.7) |
| Years after AD diagnosis | 2.5 (1.55) | 2.62 (1.59) | 2.36 (1.48) |
| Years from MCI to AD | 2.26 (1.70) | 2.26 (1.72) | 2.27 (1.69) |
| Conversion rate from MCI to AD | 266 (16%) | 150 (16%) | 116 (17%) |
| Years from AD to death | 2.28 (1.45) | 2.39 (1.48) | 2.16 (1.42) |
| Age at death | 81.7 (8.9) | 82.1 (9.4) | 81.2 (8.4) |
| Mortality rate, N (%) | 209 (13%) | 109 (11%) | 100 (14%) |
| **Hispanic, N (%)** | | | |
| Hispanic | 426 (26%) | 248 (26%) | 178 (25%) |
| Not Hispanic | 1,230 (73%) | 707 (73%) | 523 (74%) |
| No Hispanic information | 9 (1%) | 6 (1%) | 3 (1%) |
| **Race, N (%)** | | | |
| American Indian or Alaska Native | 0 (0%) | 0 (0%) | 0 (0%) |
| Asian | 10 (1%) | 5 (1%) | 5 (1%) |
| Black or African American | 259 (16%) | 157 (16%) | 102 (15%) |
| White | 1,298 (77%) | 741 (77%) | 557 (79%) |
| Multiple race | 15 (1%) | 6 (1%) | 9 (1%) |
| Unknown | 83 (5%) | 52 (5%) | 31 (4%) |
| **Vital Signs** | | | |
| Body Mass Index (BMI) | 26.5 (5.11) | 26.8 (4.21) | 26.3 (5.71) |
| **Smoking status, N (%)** | | | |
| Non-smoker | 125 (8%) | 81 (8%) | 44 (6%) |
| Current smoker | 15 (1%) | 10 (1%) | 5 (1%) |
| Ex-smoker | 87 (5%) | 37 (4%) | 50 (7%) |
| Unknown | 1**,**438 (86%) | 833 (87%) | 605 (86%) |
| **Comorbidity, N (%)** | | | |
| Neurological disorders | 606 (36%) | 346 (36%) | 260 (37%) |
| Cardiovascular diseases | 304 (18%) | 163 (17%) | 141 (20%) |
| Diabetes | 779 (47%) | 439 (45%) | 340 (48%) |

**Supplementary Table 3.** Descriptive statistics on the characteristics of sex-specific AD progression subsequence clusters (i.e., states)

| **States, N** | **C1**  **(N = 4,726)** | **C2**  **(N= 983)** | **C3**  **(N= 2,498)** | **C4**  **(N= 9,537)** | **C5**  **(N= 1,258)** | **C6**  **(N= 3,743)** | **C7**  **(N= 4,583)** |
| --- | --- | --- | --- | --- | --- | --- | --- |
| **Demographics** | | | | | | | |
| Age at AD diagnosis, mean (std) | 77.1  (9.27) | 78.1 (9.85) | 77.6 (9.54) | 77.2 (9.43) | 75.5 (9.27) | 76.1 (8.73) | 77.2  (8.35) |
| N (%) of female AD patients with a specific state, [out of N = 961] | 921  (96%) | 554 (58%) | 866 (90%) | 846 (88%) | 52  (5%) | 3  (0%) | 1  (0%) |
| N (%) of male patients with a specific state, [out of N = 704] | 704 (100%) | 18 (3%) | 4  (1%) | 0  (0%) | 514 (73%) | 681 (97%) | 531  (75%) |
| **Disease development, mean (std)** | | | | | | | |
| Follow-up durations | 8.1  (1.7) | 8.16 (1.66) | 8.23  (1.67) | 8.32  (1.65) | 7.98  (1.69) | 7.98  (1.7) | 8.17  (1.6) |
| Years before AD diagnosis | 5.59  (1.71) | 5.55 (1.72) | 5.59  (1.74) | 5.65  (1.74) | 5.56  (1.64) | 5.61  (1.7) | 5.73  (1.71) |
| Years after AD diagnosis | 2.51  (1.55) | 2.61 (1.6) | 2.64  (1.59) | 2.66  (1.58) | 2.42  (1.5) | 2.37  (1.49) | 2.45  (1.49) |
| Years from MCI to AD | 2.26  (1.7) | 2.14 (1.62) | 2.26  (1.69) | 2.29  (1.68) | 2.24  (1.66) | 2.26  (1.74) | 2.29  (1.79) |
| Conversion rate from MCI to AD | 45  (3%) | 36 (2%) | 85  (5%) | 136  (8%) | 36  (2%) | 85  (5%) | 93  (6%) |
| Years from AD to death | 2.28  (1.45) | 2.34 (1.47) | 2.34  (1.44) | 2.4  (1.5) | 2.36  (1.52) | 2.16  (1.43) | 2.18  (1.48) |
| Age at death | 81.7  (8.9) | 82.2 (9.1) | 82.1  (9.7) | 82.4  (9.3) | 80.3  (8.9) | 81.2  (8.4) | 81.5  (8.3) |
| **Hispanic, N (%) [out of N = 1,665; same for the following rows]** | | | | | | | |
| Hispanic | 426 (26%) | 142 (9%) | 225 (14%) | 223 (13%) | 141  (9%) | 173 (10%) | 132  (8%) |
| Not Hispanic | 1190 (71%) | 426 (26%) | 639 (38%) | 619 (37%) | 423 (25%) | 508 (31%) | 398 (24%) |
| No Hispanic information | 9  (1%) | 4  (0%) | 6  (0%) | 4  (0%) | 2  (0%) | 3  (0%) | 2  (0%) |
| **Race, N (%)** | | | | | | | |
| American Indian or Alaska Native | 0 (0%) | 0 (0%) | 0 (0%) | 0 (0%) | 0 (0%) | 0 (0%) | 0 (0%) |
| Asian | 10 (1%) | 3 (0%) | 4 (0%) | 5 (0%) | 5 (0%) | 5 (0%) | 4 (0%) |
| Black or African American | 259 (16%) | 83  (5%) | 139 (8%) | 143  (9%) | 83  (5%) | 102  (6%) | 86  (5%) |
| White | 1,278 (77%) | 448 (27%) | 669 (40%) | 645 (39%) | 448 (27%) | 539 (32%) | 418 (25%) |
| Multiple race | 15 (1%) | 4 (0%) | 6 (0%) | 6 (0%) | 9 (1%) | 8 (1%) | 5 (0%) |
| Unknown | 63 (4%) | 34 (2%) | 52 (3%) | 47 (3%) | 21 (1%) | 30 (2%) | 19 (1%) |
| **Vital Signs** | | | | | | | |
| Body Mass Index (BMI) | 26.5 (5.1) | 27.0 (4.3) | 26.7 (4.1) | 26.7 (4.1) | 26.3 (5.7) | 26.4 (5.9) | 26.4 (5.9) |
| **Smoking status, N (%)** | | | | | | | |
| Non-smoker | 125 (8%) | 58 (3%) | 76 (5%) | 65 (4%) | 36 (2%) | 42 (3%) | 36 (2%) |
| Current smoker | 15 (1%) | 7 (0%) | 9 (1%) | 7 (0%) | 5 (0%) | 5 (0%) | 3 (0%) |
| Ex-smoker | 87 (5%) | 24 (1%) | 36 (2%) | 33 (2%) | 45 (3%) | 46 (3%) | 34 (2%) |
| Unknown | 1,398 (84%) | 483 (29%) | 749 (45%) | 741 (45%) | 480 (29%) | 591 (35%) | 459 (28%) |
| **Comorbidity, N (%)** | | | | | | | |
| Neurological disorders | 601 (36%) | 223 (13%) | 270 (16%) | 313 (19%) | 198 (12%) | 246 (15%) | 202 (12%) |
| Cardiovascular diseases | 298 (18%) | 86 (5%) | 157 (9%) | 161 (10%) | 91 (5%) | 96 (6%) | 101 (6%) |
| Diabetes | 775 (47%) | 133 (8%) | 295 (18%) | 403 (24%) | 255 (15%) | 335 (20%) | 253 (15%) |

**Supplementary Table 4.** Descriptive statistics on the characteristics of sex-specific AD progression sub-phenotypes.

| **Sub-phenotypes, N** | **S1**  **[C1→C2→C3→C4]**  **(N = 413)** | **S2**  **[C1→C3→C4]**  **(N= 329)** | **S3**  **[C1→C5→C6→C7]**  **(N= 346)** | **S4**  **[C1→C6→C7]**  **(N= 178)** | **S5**  **[C1→C5→C6]**  **(N= 137)** |
| --- | --- | --- | --- | --- | --- |
| **Demographics** | | | | | |
| Age at AD diagnosis, mean (std) | 77.2 (9.8) | 76.9 (9.2) | 76.2 (8.7) | 77.1 (8.3) | 74.8 (9.5) |
| Female, N (%) | 406 (98%) | 326 (99%) | 1 (0%) | 6 (3%) | 2 (2%) |
| Male, N (%) | 7 (2%) | 3 (1%) | 345 (100%) | 172 (97%) | 135 (98%) |
| **Disease development, mean (std)** | | | | | |
| Follow-up durations | 8.3 (1.61) | 8.29 (1.7) | 8.24 (1.57) | 8.05 (1.67) | 7.39 (1.8) |
| Years before AD diagnosis | 5.67 (1.73) | 5.69 (1.76) | 5.76 (1.66) | 5.68 (1.8) | 5.25 (1.52) |
| Years after AD diagnosis | 2.64 (1.56) | 2.6 (1.54) | 2.48 (1.46) | 2.37 (1.52) | 2.14 (1.42) |
| Years from MCI to AD | 2.25 (1.65) | 2.23 (1.79) | 2.29 (1.8) | 2.3 (1.8) | 2.23 (1.68) |
| Conversion rate from MCI to AD | 66 (16%) | 53 (16%) | 58 (17%) | 29 (16%) | 21 (15%) |
| Years from AD to death | 2.27 (1.46) | 2.31 (1.32) | 2.26 (1.48) | 1.93 (1.51) | 2.37 (1.22) |
| Age at death | 82.9 (9.0) | 82.1 (10.1) | 80.7 (8.8) | 82.7 (7.1) | 79.6 (8.7) |
| **Hispanic, N (%)** |  |  |  |  |  |
| Hispanic | 111 (27%) | 84 (26%) | 85 (25%) | 43 (24%) | 38 (28%) |
| Not Hispanic | 300 (72%) | 243 (73%) | 260 (75%) | 134 (75%) | 98 (71%) |
| No Hispanic information | 2 (1%) | 2 (1%) | 1 (0%) | 1 (1%) | 1 (1%) |
| **Race, N (%)** |  |  |  |  |  |
| American Indian or Alaska Native | 0 (0%) | 0 (0%) | 0 (0%) | 0 (0%) | 0 (0%) |
| Asian | 3 (1%) | 0 (0%) | 3 (1%) | 1 (1%) | 1 (1%) |
| Black or African American | 56 (14%) | 60 (18%) | 55 (16%) | 26 (15%) | 12 (9%) |
| White | 328 (78%) | 250 (76%) | 279 (80%) | 139 (77%) | 110 (80%) |
| Multiple race | 2 (1%) | 2 (1%) | 3 (1%) | 2 (1%) | 3 (2%) |
| Unknown | 24 (6%) | 17 (5%) | 6 (2%) | 10 (6%) | 11 (8%) |
| **Vital Signs** | | | | | |
| Height, inch | 62.7 (4.8) | 67.1 (4.3) | 69.0 (3.1) | 69.0 (2.9) | 70.8 (3.8) |
| Weight, lbs. | 152.8 (32.6) | 154.3 (34.4) | 193.0 (40.6) | 189.1 (33.8) | 182.2 (36.4) |
| Diastolic Blood Pressure, mmHg | 72.6 (11.3) | 73.3 (12.2) | 76.2 (11.6) | 75.7 (12.3) | 74.8 (10.5) |
| Systolic Blood Pressure, mmHg | 135.8 (21.6) | 135.4 (21.4) | 134.6 (20.4) | 133.2 (17.6) | 133.6 (22.3) |
| Body Mass Index (BMI) | 26.9 (5.1) | 27.0 (5.8) | 27.9 (4.3) | 27.7 (4.6) | 27.9 (4.3) |
| **Smoking status, N (%)** | | | | | |
| Non-smoker | 42 (10%) | 30 (9%) | 24 (7%) | 9 (5%) | 8 (6%) |
| Current smoker | 5 (1%) | 1 (0%) | 3 (1%) | 2 (1%) | 1 (1%) |
| Ex-smoker | 16 (4%) | 16 (5%) | 28 (8%) | 16 (9%) | 8 (6%) |
| Unknown | 350 (85%) | 282 (86%) | 291 (84%) | 151 (85%) | 120 (87%) |
| **Comorbidity, N (%)** | | | | | |
| Neurological disorders | 149 (36%) | 113 (34%) | 143 (41%) | 70 (39%) | 37 (27%) |
| Cardiovascular diseases | 68 (16%) | 65 (20%) | 78 (23%) | 34 (19%) | 21 (15%) |
| Diabetes | 171 (41%) | 157 (48%) | 178 (51%) | 92 (52%) | 50 (36%) |

**Supplementary Table 5.** Consistency of clusters and subphenotypes across different latent dimensions (Baseline: latent dim: 16, distance metric: Euclidean, linkage method: Ward).

| **Latent dim** | **AMI of Clusters** | **ARI of Clusters** | **AMI of Subtypes** | **ARI of Subtypes** |
| --- | --- | --- | --- | --- |
| 8 | 0.789 | 0.725 | 0.695 | 0.687 |
| 32 | 0.763 | 0.758 | 0.701 | 0.697 |
| 64 | 0.754 | 0.736 | 0.692 | 0.681 |

**Supplementary Table 6.** Consistency of clusters and subphenotypes across different linkage methods (Baseline: latent dim: 16, distance metric: Euclidean, linkage method: Ward).

| **Linkage** | **AMI of Clusters** | **ARI of Clusters** | **AMI of Subtypes** | **ARI of Subtypes** |
| --- | --- | --- | --- | --- |
| Complete | 0.746 | 0.721 | 0.681 | 0.632 |
| Average | 0.856 | 0.812 | 0.711 | 0.697 |
| Centroid | 0.792 | 0.731 | 0.712 | 0.618 |

**Supplementary Table 7.** Consistency of clusters and subphenotypes across different distance metrics (Baseline: latent dim: 16, distance metric: Euclidean, linkage method: Average).

| **Distance** | **AMI of Clusters** | **ARI of Clusters** | **AMI of Subtypes** | **ARI of Subtypes** |
| --- | --- | --- | --- | --- |
| Euclidean | 0.856 | 0.812 | 0.711 | 0.697 |
| Cosine | 0.508 | 0.373 | 0.464 | 0.381 |
| Correlation | 0.926 | 0.907 | 0.854 | 0.837 |

**Supplementary Table 8.** Consistency of clusters and subphenotypes on unsupervised representation, sex-stratified unsupervised representation, and supervised latent representation (excluding strongly sex-linked features).

| **Settings** | **AMI of Clusters** | **ARI of Clusters** | **AMI of Subtypes** | **ARI of Subtypes** |
| --- | --- | --- | --- | --- |
| Unsupervised representation | 0.132 | 0.105 | 0.096 | 0.050 |
| Unsupervised representation (sex-stratified) | 0.601 | 0.528 | 0.535 | 0.402 |
| Supervised representation (excluding top 10% sex-linked features) | 0.967 | 0.944 | 0.912 | 0.901 |
| Supervised representation (excluding top 20% sex-linked features) | 0.940 | 0.921 | 0.897 | 0.889 |
| Supervised representation (excluding top 30% sex-linked features) | 0.909 | 0.866 | 0.852 | 0.830 |

**Supplementary Table 9. Cox proportional hazards model results and log-rank tests.**

|  | **Patients with Hypertension** |  | **Patients with Essential Hypertension** |  | **Patients with Hyperlipidemia** |  |
| --- | --- | --- | --- | --- | --- | --- |
| **Variable** | **HR (95% CI)** | **P-value** | **HR (95% CI)** | **P-value** | **HR (95% CI)** | **P-value** |
| Male- vs. Female- dominant subphenotypes | 1.35 (1.05–2.16) | <0.001 | 1.54 (1.17–2.39) | <0.001 | 1.21 (0.92–2.05) | <0.001 |
| *Covariates* | | | | | | |
| Age | 0.99 (0.98–1.00) | 0.02 | 0.99 (0.98–1.00) | 0.19 | 0.99 (0.98–1.00) | <0.005 |
| Hispanic | 0.94 (0.79–1.11) | 0.44 | 1.03 (0.81–1.29) | 0.83 | 1.14 (0.95–1.35) | 0.15 |
| Race: Black (ref = White) | 1.11 (0.81–1.53) | 0.51 | 0.83 (0.55–1.27) | 0.39 | 0.98 (0.71–1.37) | 0.92 |
| Race: Other (ref = White) | 1.16 (0.96–1.40) | 0.12 | 1.09 (0.87–1.37) | 0.46 | 1.11 (0.91–1.35) | 0.31 |
| No. encounters | 1.00 (1.00–1.00) | <0.001 | 1.00 (1.00–1.00) | 0.17 | 1.00 (1.00–1.00) | 0.01 |
| *Model statistics* | | | | | | |
| N (events / censored) | 1,403 (1,314 / 89) |  | 1,403 (1,294 / 109) |  | 1,403 (1,201 / 202) |  |
| PH assumption | Pass |  | Pass |  | Pass |  |
| Hazard ratios (HR) were estimated using adjusted Cox proportional hazards models. P-values for covariates are from the adjusted Cox models. Log-rank p-values compare survival curves between male- and female-dominant subphenotypes. | | | | | | |

**Supplementary Table 10. Hyperparameter settings**

| **Hyperparameter** | **Settings** |
| --- | --- |
| **Framework** | Python (v3.12), PyTorch (v2.10.0, CUDA 11.8) |
| **Hidden layers** | [256, 128, 64, 1] |
| **Subsequence window** | 6 months |
| **Input features** | Demographics + Phenotypes |
| **Batch size** | 64 |
| **Max epochs** | 500 |
| **Learning rate** | 1e-3 |
| **Dropout** | 0.2 |
| **Weight decay** | 1e-3 |
| **Early stopping patience** | 20 |
| **Classification loss weight** | 0.5 |
| **Reconstruction loss weight** | 0.5 |
| **Decision threshold** | 0.5 |

**Supplementary References**

1. Lyketsos, C. G. & Olin, J. Depression in Alzheimer’s disease: overview and treatment. *Biol. Psychiatry* **52**, 243–252 (2002).

2. Ju, Y.-E. S. *et al.* Sleep quality and preclinical Alzheimer disease. *JAMA Neurol.* **70**, 587–593 (2013).

3. Wang, C. & Holtzman, D. M. Bidirectional relationship between sleep and Alzheimer’s disease: role of amyloid, tau, and other factors. *Neuropsychopharmacology* **45**, 104–120 (2020).

4. Newman, A. B. *et al.* Dementia and Alzheimer’s disease incidence in relationship to cardiovascular disease in the Cardiovascular Health Study cohort. *J. Am. Geriatr. Soc.* **53**, 1101–1107 (2005).

5. Rosenberg, P. B. *et al.* Effects of cardiovascular medications on rate of functional decline in Alzheimer disease. *Am. J. Geriatr. Psychiatry* **16**, 883–892 (2008).

6. Wang, X.-F. *et al.* Linking Alzheimer’s disease and type 2 diabetes: Novel shared susceptibility genes detected by cFDR approach. *J. Neurol. Sci.* **380**, 262–272 (2017).

7. Janson, J. *et al.* Increased risk of type 2 diabetes in Alzheimer disease. *Diabetes* **53**, 474–481 (2004).
